# Deaths from tuberculosis: differences between tuberculosis-related and non-tuberculosis-related deaths

**DOI:** 10.3389/fpubh.2023.1207284

**Published:** 2023-09-01

**Authors:** Yun-Jeong Jeong, Jae Seuk Park, Hyung Woo Kim, Jinsoo Min, Yousang Ko, Jee Youn Oh, Eun Hye Lee, Bumhee Yang, Min Ki Lee, Yun Seong Kim, Jung Hyun Chang, Yangjin Jegal, Sung Soon Lee, Ju Sang Kim, Hyeon-Kyoung Koo

**Affiliations:** ^1^Division of Pulmonary and Critical Care Medicine, Department of Internal Medicine, Dongguk University Ilsan Hospital, Goyang, Republic of Korea; ^2^Division of Pulmonary Medicine, Department of Internal Medicine, Dankook University College of Medicine, Cheonan, Republic of Korea; ^3^Division of Pulmonary and Critical Care Medicine, Department of Internal Medicine, Incheon St. Mary's Hospital, College of Medicine, The Catholic University of Korea, Seoul, Republic of Korea; ^4^Division of Pulmonary and Critical Care Medicine, Department of Internal Medicine, Seoul St. Mary’s Hospital, College of Medicine, The Catholic University of Korea, Seoul, Republic of Korea; ^5^Division of Pulmonary, Allergy and Critical Care Medicine, Department of Internal Medicine, Kangdong Sacred Heart Hospital, Hallym University College of Medicine, Seoul, Republic of Korea; ^6^Division of Pulmonary, Allergy, and Critical Care Medicine, Department of Internal Medicine, Korea University Guro Hospital, Korea University College of Medicine, Seoul, Republic of Korea; ^7^Division of Pulmonology, Allergy and Critical Care Medicine, Department of Internal Medicine, Yongin Severance Hospital, Yonsei University College of Medicine, Seoul, Republic of Korea; ^8^Division of Pulmonary and Critical Care Medicine, Department of Internal Medicine, Chungbuk National University Hospital, Chungbuk National University College of Medicine, Cheongju, Republic of Korea; ^9^Department of Internal Medicine, Pusan National University Hospital, Pusan National University School of Medicine, Busan, Republic of Korea; ^10^Department of Internal Medicine, School of Medicine, Pusan National University Yangsan Hospital, Pusan National University and Research Institute of Convergence Biomedical Science and Technology, Busan, Republic of Korea; ^11^Division of Pulmonary and Critical Care Medicine, Department of Internal Medicine, Mokdong Hospital, Ewha Women's University, Seoul, Republic of Korea; ^12^Division of Pulmonology, Department of Internal Medicine, Ulsan University Hospital, University of Ulsan College of Medicine, Ulsan, Republic of Korea; ^13^Division of Pulmonary and Critical Care Medicine, Department of Internal Medicine, Ilsan Paik Hospital, Inje University College of Medicine, Goyang, Republic of Korea

**Keywords:** tuberculosis, death, symptom, comorbididites, cause-specific mortality, demographics

## Abstract

**Objective:**

Tuberculosis (TB) is a major cause of ill health and one of the leading causes of death worldwide. The first step in developing strategies to reduce TB mortality is to identify the direct causes of death in patients with TB and the risk factors for each cause.

**Methods:**

Data on patients with TB systemically collected from the National Surveillance System of South Korea from January 2019 to December 2020 were included in this study. We analyzed the clinical characteristics associated with TB and non-TB-related deaths, including TB-related symptoms, comorbidities, and radiographic and microbiological findings.

**Results:**

Of the total of 12,340 patients with TB, 61% were males with a mean age of 61.3 years. During the follow-up period, the overall mortality rate was 10.6%, with TB-related deaths accounting for 21.3% of all TB deaths. The median survival time in the TB-related death group was 22 days. TB-related death was associated with older age, lower body mass index (BMI), dyspnea, fever, general weakness, bilateral radiographic patterns, and acid-fast bacilli (AFB)-positive smears. Non-TB-related deaths were associated with older age, male sex, lower BMI, comorbidities of heart, liver, kidney, and central nervous system (CNS) diseases, CNS TB involvement, the presence of dyspnea, general weakness, and bilateral radiographic patterns.

**Conclusion:**

Patients with high-risk TB must be identified through cause-specific mortality analysis, and the mortality rate must be reduced through intensive monitoring of patients with a high TB burden and comorbidities.

## 1. Introduction

Before the emergence of coronavirus disease, tuberculosis (TB) was the leading cause of death from a single infectious agent, namely, *Mycobacterium tuberculosis* (1). TB was projected to cause 1.6 million deaths in 2021, an increase in number compared to 2019 and 2020 (1). Despite the end-TB strategy goal of 35% reduction between 2015 and 2020, the net reduction was only 5.9% during such period (1), and the global case fatality rate in 2020 remained high at 15%. While effective therapeutic agents are available, TB mortality remains at a standstill, and an understanding of the specific causes of TB-related mortality is essential for dealing with this situation.

TB deaths are defined as those occurring during the treatment for TB, regardless of the cause (2). Therefore, most studies utilize all-cause mortality as an indicator of TB-related mortality (3–5). However, identification of the direct causes of death and the risk factors for each cause is the first step for developing strategies to reduce TB-related mortality. South Korea has an intermediate TB burden (59.0 per 100,000 people in 2019) and a low human immunodeficiency virus (HIV) prevalence (<0.1%) (6). The risk factors for TB deaths tend to vary depending on HIV prevalence (7). In HIV-endemic regions, HIV positivity, advanced immunosuppression, smear-negative disease, and malnutrition are risk factors for TB death (7). However, in regions with low HIV prevalence, the risk factors include older age, male sex, multiple comorbidities, smear-positive disease, and alcohol and substance abuse (7–12).

Most studies attempting to determine the actual causes of death during TB treatment relied on statistics from registries or death certificates and reported TB-related death rates ranging from 10 to 50.5% (13–16). However, most registries do not contain clinical or microbiological information, and death certificates may not reflect the actual causes of death due to reporting bias. Thus, improving TB outcomes by targeting risk groups requires a deeper understanding of the actual causes of death and the influence of comorbidities on the survival of patients with TB. Therefore, we conducted this study to describe the causes and identify the risk factors for death among patients with TB in a national TB cohort in South Korea. In addition, we attempted to predict the likelihood of TB- and non-TB-related mortality.

## 2. Materials and methods

### 2.1. Study participants

In South Korea, all physicians are required to report the diagnosis and treatment of TB by the time of first suspicion or diagnosis of the disease. Under the national public–private mix (PPM) TB control project, all patients affected by the disease are monitored and reported by TB nurse specialists at the PPM participating hospitals until treatment completion (17). Data on patients diagnosed with TB between January 2019 and December 2020 were collected from PPM hospitals. The exclusion criteria were (1) patients with a change in diagnosis and (2) patients lacking information regarding treatment outcome or cause of death. Baseline characteristics of the patients such as age, sex, body mass index (BMI), smoking and alcohol history, TB-related symptoms, including cough/phlegm, dyspnea, chest pain, hemoptysis, fever, general weakness, and weight loss, previous history of TB, and comorbidities were evaluated. The comorbidities assessed included the following: lung diseases, including asthma, chronic obstructive pulmonary disease, interstitial lung disease, bronchiectasis, and pneumoconiosis; heart diseases, including angina, myocardial infarction, valvular heart disease, and heart failure; liver diseases, including liver cirrhosis, hepatocellular carcinoma, and chronic viral hepatitis; kidney diseases, including chronic renal failure (glomerular fraction rate ≤ 30 mL/min/1.73m^2^ or serum creatinine ≥2.0 mg/dL), glomerulonephritis, kidney transplant recipients, and patients undergoing hemodialysis or peritoneal dialysis; and central nervous system (CNS) diseases, including history of stroke or cerebral hemorrhage, epilepsy, Parkinson’s disease, and dementia. In addition, the results of radiographic and microbiological tests, such as sputum acid-fast bacilli (AFB) smear and culture, were collected. The presence or absence of cavitary lesions was determined based on the results of chest radiographies or computed tomographies. During follow-up assessments, treatment outcomes, including death and the time to death for deceased patients, were also documented.

### 2.2. Definitions of death

In this study, TB death was defined as all-cause mortality that occurred from the date of TB diagnosis and before completing anti-TB treatment. All TB death cases were classified as TB-related or non-TB-related deaths by the attending physicians and TB nurse specialists in each hospital. TB-related deaths were defined as cases in which TB was either the primary or a significant contributing cause of death for deceased patients, which was judged based on the underlying causes listed in the death certificate filled by the attending physician and the medical records of each patient, as well as the absence of other equally probable causes of death. In contrast, non-TB-related deaths included cases of deceased patients with TB for whom such diseases were not cited as the cause of death.

### 2.3. Ethical approval

This study was conducted in accordance with the principles of the Declaration of Helsinki. The Institutional Review Board of the Ilsan Paik Hospital, Inje University approved the study protocol (IRB no. ISPAIK 2021–08-012) and waived the need for obtaining informed consent owing to the absence of risk imposed by the study for the participants. The Korea Disease Control and Prevention Agency (KDCA) has the authority to hold and analyze surveillance data for public health and research purposes; the agency approved the use of data and provided such without personally identifiable information from the patients.

### 2.4. Statistical analysis

The characteristics of the participants are presented as means and standard deviations for continuous variables and as relative frequencies for categorical variables. Continuous variables were compared using the t-test or analysis of variance (ANOVA), and categorical variables were compared using the chi-squared test or Fisher’s exact test. All statistical analyses were performed using the R software (version 4.2.1). A correlation network was constructed using Pearson’s correlation values; each variable was represented as a specific node; node size indicated the prevalence; and links between nodes indicated significant associations (*p* < 0.05), with blue and pink colors indicating positive and negative correlations, respectively. The igraph package was used to visualize the correlation networks. Multivariable logistic regression for the development of death was performed with age, sex, BMI, and other variables with *p* < 0.1 in the univariable analysis; the best model was selected using the backward elimination method.

## 3. Results

### 3.1. Baseline characteristics

**Figure 1:**
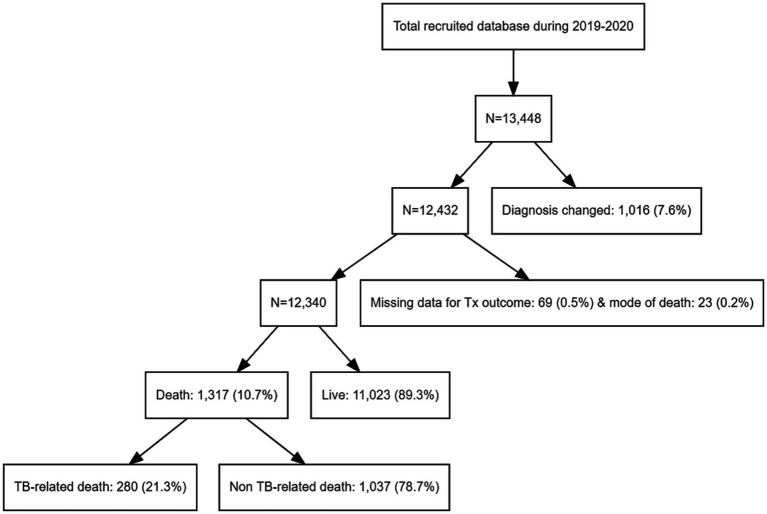
Flow chart for the study population.

Among the 13,448 patients registered between January 2019 and December 2020, 12,340 were enrolled in our study (Figure 1). The mean age of patients was 61.3 ± 19.0 years, and 7,540 (61.1%) were male, with a male to female ratio of 1.28. The study population included 8,760 (70.9%), 2,663 (21.6%), and 917 (7.4%) cases of pulmonary TB, extrapulmonary TB (EPTB), and both pulmonary and EPTB, respectively. Overall, 1,317 (10.6%) patients died during the follow-up period. The characteristics of the deceased patients and of those who survived are summarized in Table 1. Patients who died were older (59.9 ± 18.9 years vs. 73.5 ± 15.1 years, *p* < 0.001) and had a lower BMI (21.7 ± 3.4 vs. 20.4 ± 3.7, p < 0.001). Comorbidities such as diabetes, lung, heart, liver, kidney, and CNS diseases were more prevalent among the deceased patients (57.8% vs. 81.5%, p < 0.001). The proportion of EPTB cases was lower in the death group; however, among cases showing extrapulmonary involvement, TB pleurisy and CNS TB were more prevalent in deceased patients. Among the 1,684 patients with TB pleurisy, 616 (36.6%) had pulmonary involvement as well, while 1,068 (53.4%) did not. TB pleurisy was confirmed bacteriologically in 22.1% of cases, and the remaining cases were clinically diagnosed in the case of exudative pleural effusion with elevated adenosine deaminase and exclusion of parapneumonic effusion and malignancy by chest computed tomography (CT) scan and cytology. In contrast, TB lymphadenopathy (LNP), bone/joint TB, and urogenital TB were less prevalent. Dyspnea (15.7% vs. 32.7%, *p* < 0.001), fever (12.7% vs. 16.5%, p < 0.001), and general weakness (4.3% vs. 13.2%, p < 0.001) were more prevalent in the death group, whereas cough/phlegm, chest pain, and hemoptysis were less prevalent. The occurrence of weight loss did not differ significantly between the groups. In radiographic and microbiological assessments, the patients who died were more likely to have bilateral lesions and AFB-positive smears and cultures. Resistance to isoniazid was lower in deceased patients (2.5% vs. 1.4%, *p* = 0.014), whereas that to rifampin did not differ significantly (0.6% vs. 0.7%, *p* = 733). Mortality was higher in patients aged over 70 years (18.7%), underweight patients (BMI < 18.5 kg/m^2^, 18.5%), those with comorbid diseases of the heart (19.8%), liver (16.1%), kidney (26.4%), or CNS (23.3%), those with CNS involvement of TB (16.6%), and patients showing dyspnea (20.0%), fever (13.4%), general weakness (26.6%), bilateral lesions (16.0%), and AFB-positive smears (15.5%) and cultures (12.8%). The death rate stratified by cause of death according to the presence of risk factors is summarized in Table 2. The mean survival of the deceased patients was 83.3 ± 91.3 days.

**Table 1:** Baseline characteristics of the study population.

		Survived (*N* = 11,023)	Death (*N* = 1,317)				*p*-value
			Total	TB-related (*N* = 280)	Non-TB-related (*N* = 1,037)	*p*-value	
Age (years)		59.9 ± 18.9	73.5 ± 15.1	73.3 ± 16.6	73.6 ± 14.7	0.823	<0.001
Male sex (*n*, %)		6,707 (60.8%)	833 (63.2%)	160 (57.1)	673 (64.9%)	0.02	0.097
Body mass index (kg/m^2^)		21.7 ± 3.4	20.4 ± 3.7	20.1 ± 3.7	20.5 ± 3.6	0.109	<0.001
Marriage (*n*, %)		8,295 (75.3%)	1,131 (85.9%)	231 (82.5%)	900 (868%)	0.227	<0.001
Family (*n*, %)		6,567 (59.6%)	668 (50.7%)	141 (50.4%)	527 (50.8%)	0.291	<0.001
Occupation (*n*, %)		2,926 (26.5%)	84 (6.4%)	14 (5.0%)	70 (6.8%)	0.408	<0.001
Current smoking (*n*, %)		2,321 (21.1%)	171 (13.0%)	37 (13.2%)	134 (12.9%)	0.977	<0.001
Heavy drinker (*n*, %)		788 (7.1%)	109 (8.3%)	30 (10.7%)	79 (7.6%)	0.122	0.152
Comorbidities (*n*, %)		6,369 (57.8%)	1,074 (81.5%)	204 (72.9%)	870 (83.9%)	<0.001	<0.001
	Diabetes	2,192 (19.9%)	371 (28.2%)	83 (29.6%)	288 (27.8%)	0.587	<0.001
	Lung disease	578 (5.2%)	109 (8.3%)	23 (8.2%)	86 (8.3%)	>0.999	<0.001
	Heart disease	592 (5.4%)	146 (11.1%)	17 (6.1%)	129 (12.4%)	0.004	<0.001
	Liver disease	261 (2.4%)	50 (3.8%)	3 (1.1%)	47 (4.5%)	0.012	0.002
	Kidney disease	368 (3.3%)	132 (10.0%)	13 (4.6%)	119 (11.5%)	0.001	<0.001
	CNS disease	953 (8.6%)	290 (22.0%)	59 (21.1%)	231 (22.3%)	0.726	<0.001
Involved site (*n*, %)							
	TB pleurisy	1,472 (45.4%)	212 (62.5%)	40 (64.5%)	172 (62.1%)	0.833	<0.001
	TB LNP	689 (21.2%)	29 (8.6%)	5 (8.1%)	24 (8.7%)	>0.999	<0.001
	GI TB	429 (13.2%)	33 (9.7%)	7 (11.3%)	26 (9.4%)	0.826	0.082
	Bone/joint TB	254 (7.8%)	15 (4.4%)	1 (1.6%)	14 (5.1%)	0.396	0.031
	CNS TB	146 (4.5%)	29 (8.6%)	7 (11.3%)	22 (7.9%)	0.548	0.002
	Urogenital TB	90 (0.8%)	2 (0.6%)	0 (0%)	2 (0.7%)	>0.999	0.025
Symptom presence (*n*, %)							
	Cough/phlegm	4,026 (36.5%)	404 (30.7%)	96 (34.3%)	308 (29.7%)	0.161	<0.001
	Dyspnea	1728 (15.7%)	431 (32.7%)	108 (38.6%)	323 (31.1%)	0.023	<0.001
	Chest pain	846 (7.7%)	65 (4.9%)	12 (4.3%)	53 (5.1%)	0.682	<0.001
	Hemoptysis	444 (4.0%)	37 (2.8%)	6 (2.1%)	31 (3.0%)	0.578	0.037
	Fever	1,399 (12.7%)	217 (16.5%)	60 (21.4%)	157 (15.1%)	0.015	<0.001
	G/W	479 (4.3%)	174 (13.2%)	52 (18.6%)	122 (11.8%)	0.004	<0.001
	Weight loss	813 (7.4%)	87 (6.6%)	25 (8.9%)	62 (6.0%)	0.104	0.338
TB classification (*n*, %)						0.063	0.001
	PTB only	7,778 (70.5%)	987 (74.3%)	218 (77.9%)	760 (73.3%)		
	PTB + EPTB	806 (7.3%)	111 (8.4%)	28 (10.0%)	83 (8.0%)		
	EPTB only	2,437 (22.1%)	227 (17.2%)	34 (12.1%)	193 (18.6%)		
Radiographic characteristics (*n*, %)							
	Cavitary disease	1,367 (12.4%)	150 (11.4%)	47 (16.8%)	103 (9.9%)	0.002	0.311
	Bilateral disease	2,711 (24.6%)	519 (39.4%)	138 (49.3%)	381 (36.7%)	<0.001	<0.001
Microbiologic characteristics (*n*, %)							
	AFB smear positivity	2,148 (19.5%)	393 (29.8%)	143 (51.1%)	250 (24.1%)	<0.001	<0.001
	AFB culture positivity	4,493 (40.7%)	660 (50.1%)	182 (65.0%)	478 (46.1%)	<0.001	<0.001
Drug susceptibility testing (*n*, %)							
	INH resistance	281 (2.5%)	19 (1.4%)	3 (1.1%)	16 (1.5%)	0.795	0.014
	RFP resistance	69 (0.6%)	10 (0.7%)	1 (0.4%)	9 (0.8%)	0.649	0.733
	Any resistance	692 (6.3%)	58 (4.3%)	12 (4.3%)	46 (4.3%)	0.861	<0.001
Follow-up period (days)		206.3 ± 92.7	83.3 ± 91.3	56.4 ± 83.0	90.6 ± 92.1	<0.001	<0.001

TB, tuberculosis; LNP, lymphadenopathy; GI, gastrointestinal; CNS, central nervous system; G/W, general weakness; PTB, pulmonary tuberculosis; EPTB, extrapulmonary tuberculosis, AFB, acid-fast bacilli; DST, drug susceptibility test; INH, isoniazid; RFP, rifampin.

**Table 2:** Death rate according to the presence of risk factors.

Mortality	Total death	TB-related death	Non-TB-related death
Total population	1,317 (10.6%)	280 (2.3%)	1,037 (8.4%)
Older adult (≥70 years)	909 (18.7%)*	192 (3.9%)*	719 (14.8%)*
Male sex	833 (11.0%)	160 (2.1%)	673 (8.9%)*
Underweight	402 (18.5%)*	100 (4.6%)*	302 (13.9%)*
Heart disease	146 (19.8%)*	17 (2.3%)	129 (17.5%)*
Liver disease	50 (16.1%)*	3 (1.0%)	47 (15.1%)*
Kidney disease	132 (26.4%)*	13 (2.6%)	119 (23.8%)*
CNS disease	290 (23.3%)*	59 (4.7%)*	231 (18.6%)*
CNS TB involvement	29 (16.6%)*	7 (4.0%)	22 (12.6%)
Dyspnea	431 (20.0%)*	108 (5.0%)	323 (15.0%)*
Fever	217 (13.4%)*	60 (3.7%)*	157 (9.7%)*
General weakness	174 (26.6%)*	52 (8.0%)*	122 (18.7%)*
Bilateral disease	519 (16.0%)*	138 (4.3%)*	381 (11.7%)*
AFB smear +	393 (15.5%)*	143 (5.6%)*	250 (9.9%)*
AFB culture +	660 (12.8%)*	182 (3.5%)*	478 (9.3%)*

* statistically significant difference.

TB, tuberculosis; CNS, central nervous system; AFB, acid-fast bacilli.

### 3.2. Comparisons of TB- and non-TB-related mortality

Among the deceased patients, 280 (21.3%) died of TB-related causes and 1,037 (78.7%) died of non-TB-related causes. The characteristics of these patients are compared in Table 1. Age did not differ significantly between the two groups (73.3 ± 16.6 vs. 73.6 ± 14.7 years, *p* = 0.823).

**Figure 2:**
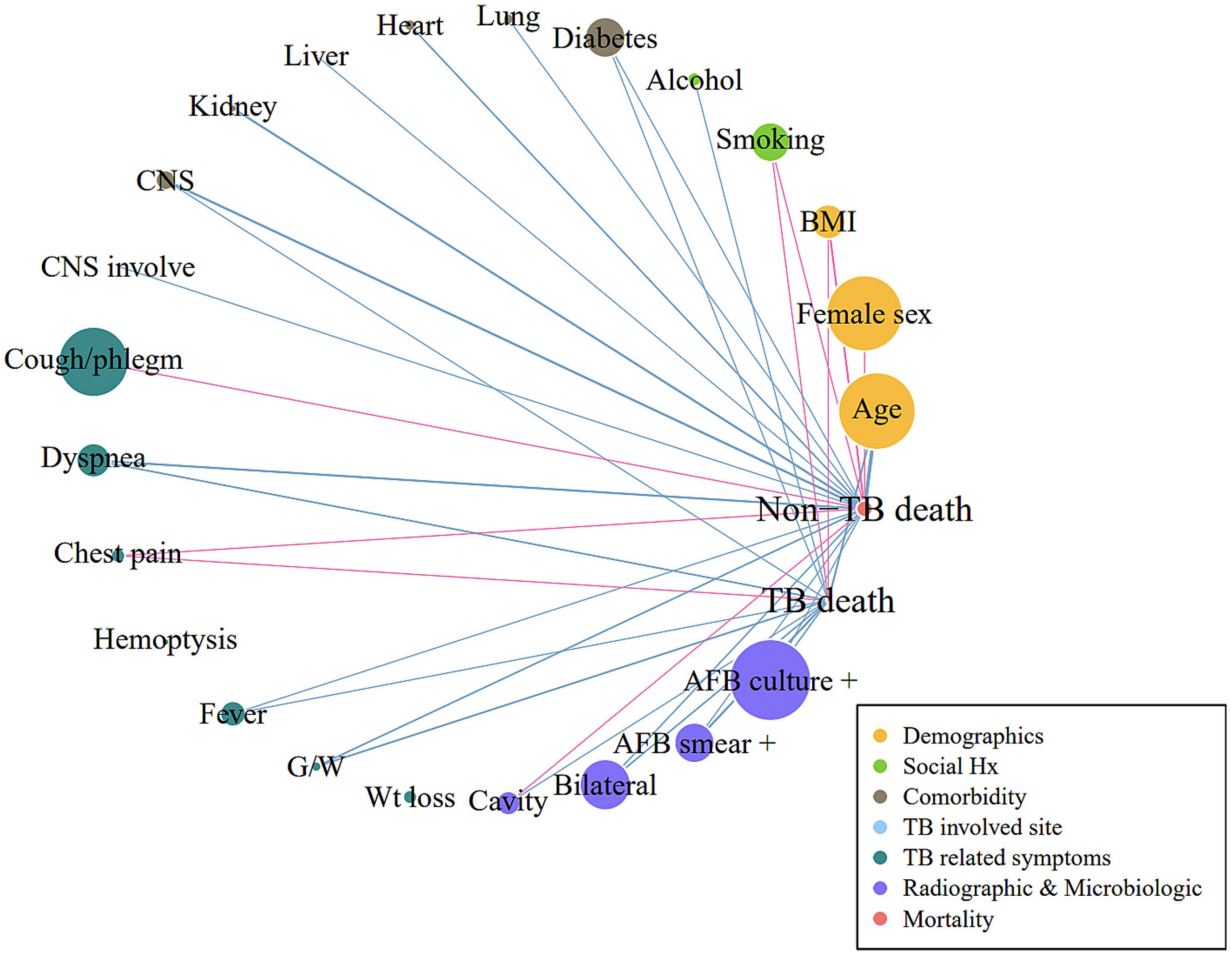
Correlation network for TB-related and non-TB-related deaths Pearson’s correlation analysis between variables was performed. Links or edges between nodes indicate statistically significant associations (*p* < 0.05). The thickness of the edges was correlated with the strength of their associations (Pearson’s *R* coefficient): blue and pink indicate positive and negative correlations, respectively. BMI, body mass index; CNS, central nervous system; G/W, general weakness; AFB, acid-fast bacilli; TB, tuberculosis.

The TB-related death group was more likely to exhibit dyspnea (38.6% vs. 31.1%, *p* = 0.023), fever (21.4% vs. 15.1%, *p* = 0.015), and general weakness (18.6% vs. 11.8%, *p* = 0.004). Radiographic and microbiological assessments showed that the rates of cavitary lesions (16.8% vs. 9.9%, *p* = 0.002), bilateral lesions (49.3% vs. 36.7%, *p* < 0.001), AFB smear positivity (51.1% vs. 24.1%, p < 0.001), and AFB culture positivity (65.0% vs. 46.1%, p < 0.001) were all higher in the TB-related death group. The non-TB-related death group included a higher proportion of male patients (57.1% vs. 64.9%, *p* = 0.020) and a greater prevalence of comorbidities, including heart (6.1% vs. 12.4%, *p* = 0.004), liver (6.1% vs. 12.4%, *p* = 0.012), and kidney disease (4.6% vs. 11.5%, *p* = 0.001). The proportion of patients with EPTB was lower among those who died of TB. A correlation network demonstrating the interrelationships between TB- and non-TB-related deaths is shown in Figure 2.

**Figure 3:**
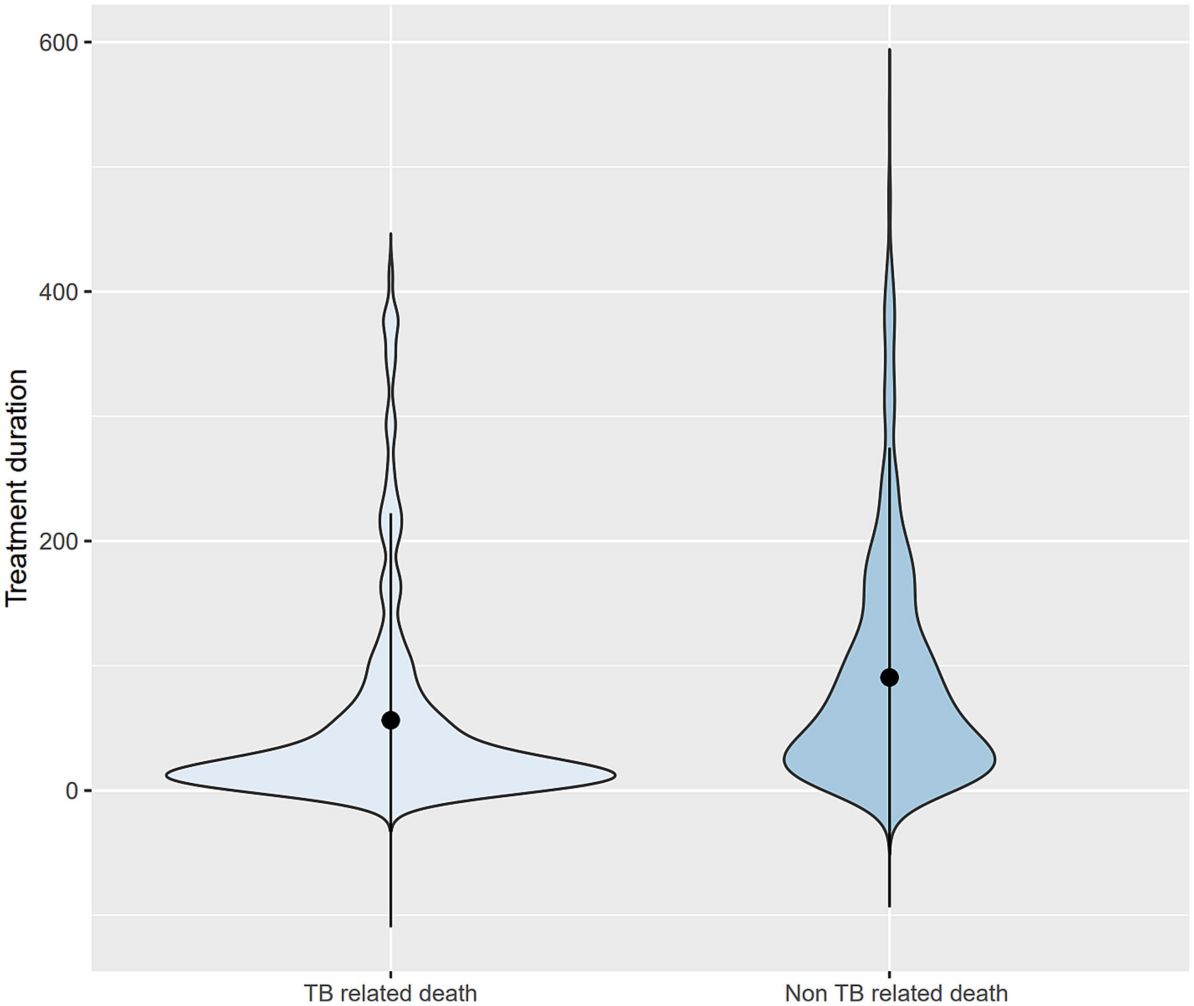
Violin plots for treatment duration comparing TB- and non-TB-related deaths. TB, tuberculosis.

The survival time was shorter in the TB-related death group (56.4 ± 83.0 days) than in the non-TB-related death group (90.6 ± 92.1 days). The distribution of treatment duration for TB-related and non-TB-related deaths was right-skewed (Figure 3). The median survival times were estimated to be 22 days (interquartile range [IQR], 9–59 days) and 60.5 days (IQR, 24.75–126 days) in the TB and non-TB-related death groups, respectively, indicating that half of the deceased patients died within 1–2 months of treatment initiation.

### 3.3. Model for prediction of death

Logistic regression was performed to predict the risk of death for TB-related and non-TB-related causes. Table 3 summarizes the results of the univariable analyses for TB and non-TB-related deaths. In multivariable analyses, all-cause death was independently associated with older age (Odds Ratio (OR) = 1.04, 95% Confidence Interval (CI) = 1.04–1.05), male sex (OR = 1.22, 95% CI = 1.08–1.41), lower BMI (OR = 0.91, 95% CI = 0.89–0.93), heavy drinking (OR = 1.28, OR = 1.01–1.61), heart disease (OR = 1.25, 95% CI = 1.02–1.54), liver disease (OR = 1.66, 95% CI = 1.19–2.32), kidney disease (OR = 2.32, 95% CI = 1.85–2.90), CNS disease (OR = 1.57, 95% CI = 1.33–1.84), CNS TB involvement (OR = 3.56, 95% CI = 2.31–5.48), dyspnea (OR = 1.58, 95% CI = 1.63–2.08), general weakness (OR = 1.96, 95% CI = 1.60–2.39), weight loss (OR = 0.70, 95% CI = 0.55–0.90), bilateral lesions (OR = 1.39, 95% CI = 1.22–1.58), and AFB smear positivity (OR = 1.41, 95% CI = 1.23–1.63).

**Table 3:** UnivaPriable and multivariable analysis for (A) TB-related and (B) non-TB-related deaths.

(A)				
	Univariable		Multivariable	
	OR	95% CI	OR	95% CI
Age	1.044	1.035–1.053	1.035	1.027–1.044
Body mass index	0.869	0.837–0.901	0.930	0.896–0.965
Current smoking	0.596	0.420–0.844	—	—
Heavy drinker	1.549	1.054–2.277	—	—
Diabetes	1.628	1.252–2.111	—	—
Lung disease	1.536	0.995–2.369	—	—
CNS disease	2.452	1.829–3.289	—	—
Dyspnea	3.064	2.398–3.916	2.110	1.633–2.727
Fever	1.841	1.377–2.461	1.581	1.172–2.134
General weakness	4.349	3.183–5.942	2.462	1.774–3.416
Cavitary lesion	1.453	1.057–1.997	—	—
Bilateral lesion	2.82	2.223–3.577	1.728	1.345–2.221
AFB smear positivity	4.206	3.313–5.339	2.932	2.282–3.769
AFB culture positivity	2.649	2.067–3.396	—	—

(B)				
	Univariable		Multivariable	
	OR	95% CI	OR	95% CI
Age	1.049	1.044–1.053	1.041	1.036–1.046
Male sex	1.195	1.046–1.364	1.382	1.197–1.595
Body mass index	0.896	0.879–0.914	0.912	0.894–0.931
Current smoking	0.563	0.467–0.679	—	—
Diabetes	1.526	1.332–1.761	—	—
Lung disease	1.610	1.272–2.037	—	—
Heart disease	2.495	2.039–3.052	1.409	1.134–1.752
Liver disease	1.987	1.447–2.728	2.147	1.532–3.009
Kidney disease	3.716	2.992–4.615	2.574	2.042–3.246
CNS disease	2.914	2.484–3.420	1.483	1.245–1.766
TB pleurisy	1.288	1.084–1.530	—	—
CNS TB	1.580	1.006–2.481	2.854	1.762–4.621
Cough/phlegm	0.736	0.641–0.845	—	—
Dyspnea	2.333	2.027–2.685	1.589	1.367–1.848
Chest pain	0.656	0.493–0.872	—	—
Fever	1.204	1.001–1.439	—	—
General weakness	2.705	2.197–3.330	1.620	1.293–2.030
Weight loss	0.794	0.609–1.036	0.732	0.552–0.970
Cavitary lesion	0.771	0.625–0.952	—	—
Bilateral lesion	1.724	1.509–1.970	1.261	1.093–1.454
AFB smear positivity	1.250	1.076–1.451	—	—
AFB culture positivity	1.213	1.067–1.378	—	—

OR, odds ratio; CI, confidential interval; CNS, central nervous system; TB, tuberculosis; AFB, acid-fast bacilli.

TB-related death was independently associated with older age (OR = 1.035, 95% CI = 1.027–1.044), lower BMI (OR = 0.930, 95% CI = 0.896–0.965), and the presence of dyspnea (OR = 2.110, 95% CI = 1.633–2.727), fever (OR = 1.581, 95% CI = 1.172–2.134), general weakness (OR = 2.463, 95% CI = 1.774–3.416), bilateral lesions (OR = 1.728, 95% CI = 1.345–2.221), and AFB smear positivity (OR = 2.282–3.769) (Table 3A). Non-TB-related deaths were associated with older age (OR = 1.041, 95% CI = 1.036–1.046), male sex (OR = 1.392, 95% CI = 1.197–1.595), lower BMI (OR = 0.912, 95% CI = 0.894–0.931), heart disease (OR = 1.409, 95% CI = 1.134–1.752), liver disease (OR = 2.147, 95% CI = 1.532–3.009), kidney disease (OR = 2.574, 95% CI = 2.042–3.246), CNS disease (OR = 1.483, 95% CI = 1.245–1.766), CNS TB involvement (OR = 2.854, 95% CI = 1.762–4.621), the presence of dyspnea (OR = 1.589, 95% CI = 1.367–1.848), general weakness (OR = 1.620, 95% CI = 1.293–2.030), weight loss (OR = 0.732, 95% CI = 0.552–0.970), and bilateral lesions (OR = 1.261, 95% CI = 1.093–1.454) (Table 3B). The probability of death equations for all-cause, TB-related and non-TB-related deaths are presented in Supplementary Table S1.

## 4. Discussion

The clinical characteristics associated with TB-related and non-TB-related deaths, including comorbidities, symptoms, and microbiologic burden, were analyzed using a nationwide registry cohort. Our study revealed that the overall mortality rate of TB patients during the treatment course was 10.6% and that TB-related deaths accounted for 21.3% of all deaths. Older age, lower BMI, dyspnea, general weakness, and bilateral lesions were common risk factors for both TB-related and non-TB-related deaths. While fever and AFB smear positivity were independent risk factors in the TB-related death group, male sex, comorbidities of heart, liver, kidney and CNS diseases, and CNS TB involvement were risk factors in the non-TB-related death group. Considering that older age, lower BMI, dyspnea, fever, general weakness, bilateral lesions, and positive AFB smear were significant risk factors for TB-related death, when an underweight older adult patient with fever, dyspnea, and general weakness visits the clinic, TB should be suspected, and immediate examination and treatment should be considered. Preventing delays in diagnosis and treatment for patients at high risk of death is critical for reducing TB mortality. In addition, patients with TB presenting with bilateral lesions and positive AFB smears should also be considered candidates for intensive surveillance. Since TB-related death occurred early in the treatment course, patients with TB with risk factors require intensive monitoring, such as hospitalization or short-term outpatient follow-up, particularly in the early stages of treatment. Comorbidities such as heart, liver, and kidney diseases were associated with non-TB-related deaths; therefore, comprehensive care is required for patients with TB with these underlying diseases. Our findings may contribute to a better understanding of cause-specific TB mortality and the identification of patients with TB at risk of death.

Compared to studies conducted in Canada (18) and Taiwan (19), our study found a TB-related mortality rate of 2.26%. However, TB-related mortality rates in South Africa (7%), Australia (8.7%), and Russia (5.9–6.3%) were higher than that in our findings, potentially due to poor access to healthcare or higher rates of drug-resistant TB. In addition, 78.7% of the deceased patients with TB in our study died from causes other than TB. Aging and underlying comorbidities have been identified as risk factors for all TB deaths in both developed and developing countries (15, 19–22). In our study, comorbidities such as heart, liver, and kidney diseases were specific causes of non-TB-related mortality.

Several studies have reported relationships between symptom duration and mortality risk. Prolonged symptoms were associated with an increased risk of death in studies from Russia (23) and France (24) and a decreased risk of death in studies from Singapore (9), Taiwan (25), and Malawi (26). However, to our knowledge, few studies have examined the relationship between specific TB-related symptoms and mortality. We found that dyspnea, fever, and general weakness were more prevalent in deceased patients with TB, indicating that these risk groups required close follow-up.

Notably, weight loss showed protective effects against non-TB-related mortality in this study. Of the survivors, 7.4% experienced weight loss, compared to 6.6% of all deaths. Among those in the non-TB-related deaths group and the TB-related deaths group, 6.0 and 8.9%, respectively, experienced weight loss, indicating that weight loss reduces the risk of non-TB-related death. Patients who experienced weight loss were younger (51.6 ± 19.1 vs. 58.7 ± 17.3 years, *p* ≤ 0.001); therefore, the overall prevalence of comorbid diseases, including lung (3.3% vs. 5.7%, *p* = 0.003), heart (3.3% vs. 6.1%, *p* = 0.052), kidney (2.4% vs. 4.2%, *p* = 0.014), and CNS disease (5.0% vs. 10.5%, p ≤ 0.001), was lower in patients who lost weight, except for diabetes (26.0% vs. 20.4%, p ≤ 0.001). Since non-TB-related deaths were associated with advanced age and various comorbidities (Table 3B), these variables could account for the effect of weight loss, even after adjustment. However, further studies are required to understand the underlying pathophysiology.

Among radiographic and microbiological characteristics, bilateral lesions and AFB-positive smears and cultures were more likely to be present in deceased patients. AFB positivity was associated with TB-related death but not with non-TB-related death, confirming that it is an indicator that reflects the severity of TB in regions with low HIV prevalence, such as South Korea. Bacterial confirmation by Ziehl-Neelsen staining or mycobacterial culture was shown to be a mortality risk factor in regions with low HIV prevalence (9, 16), whereas smear-negative disease is associated with a poor prognosis in regions with high HIV prevalence (27).

We observed that TB pleurisy and CNS TB were more prevalent in deceased patients with extrapulmonary involvement. Extrapulmonary involvement was more prevalent in all TB deaths and TB-related-death groups in a Taiwanese study (19) and was a significant predictor of mortality in a Dutch study (22). However, in our study, the proportion of EPTB patients was lower in the TB-related-death group, potentially due to the differences in the proportion of EPTB subtypes among the study populations. TB pleurisy accounted for a relatively higher proportion of EPTB cases in the death group (62.5%) than in the survivor group (45.4%). Whereas TB LNP was present in 8.6% of patients in the death group and 21.2% of those in the survivor group, indicating that many patients with TB LNP in the survivor group had favorable prognosis. Seven (11.3%) of 66 patients with EBTB in the TB-related-death group presented with CNS TB; therefore, it was difficult to demonstrate a statistically significant relationship due to the small sample size, and additional research is needed. A limitation of our study was that it was difficult to determine the specific cause of non-TB-related death in deceased CNS TB patients.

The key strength of our study was the large number of notified TB cases collected systemically across the country, which represents the actual burden of TB in South Korea. In addition, our data included EPTB and drug-resistant TB, reflecting the overall TB burden. Therefore, the current study can serve as a guide for the planning and development of government healthcare policies.

This study had several other potential limitations that must be recognized. First, our data relied on reports from clinicians rather than direct observations by current investigators, which may have led to inaccuracies. Second, several prominent predictors, including HIV infection, duration of treatment delay, education level, and income, could not be analyzed due to missing data. However, we believe that the methodical approach to data collection, supported by TB nurse specialists as part of the national PPM TB control project, minimized the bias in this study.

In conclusion, even in settings with adequate resources and effective therapy, the mortality rate associated with TB remains high. To improve outcomes, a deeper understanding of cause-specific mortality and individualized monitoring based on each risk factor are required.

## Data availability statement

The Korea Disease Control and Prevention Agency (KDCA) has the authority to hold and analyze surveillance data for public health and research purposes. Data are available upon reasonable request and permission from KDCA.

## Author contributions

Y-JJ, JSP, JSK, and H-KK conceived of the analysis. Y-JJ and H-KK performed all analysis, drafted the manuscript. SSL, JSK, and JSP supervised the research project. HWK, JM, YK, JYO, Y-JJ, EHL, and BY provided substantive review and edited the manuscript. Y-JJ, JSP, H-KK, JM, YK, JYO, EHL, BY, MKL, YSK, JHC, YJ, SSL, JSK, and H-KK provided data collection. All authors read and approved the final manuscript.

## Funding

The PPM TB control project is supported by the National Health Promotion Fund, funded by the KDCA, Republic of Korea; and this study was also supported by the National Research Foundation (NRF) ministry of science and ICT, Republic of Korea (2021R1G1A1095110).

## Conflict of interest

The authors declare that the research was conducted in the absence of any commercial or financial relationships that could be construed as a potential conflict of interest.

## Publisher’s note

All claims expressed in this article are solely those of the authors and do not necessarily represent those of their affiliated organizations, or those of the publisher, the editors and the reviewers. Any product that may be evaluated in this article, or claim that may be made by its manufacturer, is not guaranteed or endorsed by the publisher.

## References

[1] World Health Organization. Global Tuberculosis Report (2021). Available at: https://apps.who.int/iris/handle/10665/346387 [Accessed May 31, 2023].

[2] World Health Organization. WHO tuberculosis Programme: framework for effective tuberculosis control (1994). WHO/TB/94.179. Available at: https://apps.who.int/iris/handle/10665/58717 (accessed May 31, 2023).

[3] Oursler KK Moore RD Bishai WR Harrington SM Pope DS Chaisson RE. Survival of patients with pulmonary tuberculosis: clinical and molecular epidemiologic factors. Clin Infect Dis. (2002) 34:752–9. doi: 10.1086/338784 11850859

[4] Borgdorff MW Veen J Kalisvaart NA Nagelkerke N. Mortality among tuberculosis patients in the Netherlands in the period 1993-1995. Eur Resp J. (1998) 11:816–10. doi: 10.1183/09031936.98.11040816 9623682

[5] Hansel NN Merriman B Haponik EF Diette GB. Hospitalizations for tuberculosis in the United States in 2000: predictors of in-hospital mortality. Chest. (2004) 126:1079–86. doi: 10.1378/chest.126.4.1079, PMID: 15486367

[6] Korea centers for disease control and prevention. Annual report on the notified tuberculosis in Korea, 2019. Cheongju, Korea: Korea Centers for Disease Control and Prevention (2020).

[7] Waitt CJ Squire SB. A systematic review of risk factors for death in adults during and after tuberculosis treatment. Int J Tuberc Lung Dis. (2011) 15:871–5. doi: 10.5588/ijtld.10.0352, PMID: 21496360

[8] Lefebvre N Falzon D. Risk factors for death among tuberculosis cases: analysis of European surveillance data. Eur Resp J. (2008) 31:1256–60. doi: 10.1183/09031936.00131107, PMID: 18515556

[9] Low S Ang LW Cutter J James L Chee CBE Wang Y. Mortality among tuberculosis patients on treatment in Singapore. Int J Tuberc Lung Dis. (2009) 13:328–4. PMID: 19275792

[10] Walpola HC Siskind V Patel AM Konstantinos A Derhy P. Tuberculosis-related deaths in Queensland, Australia, 1989–1998: characteristics and risk factors. Int J Tuberc Lung Dis. (2003) 7:742–50. PMID: 12921150

[11] Horita N Miyazawa N Yoshiyama T Sato T Yamamoto M Tomaru K. Development and validation of a tuberculosis prognostic score for smear-positive in-patients in Japan. Int J Tuberc Lung Dis. (2013) 17:54–60. doi: 10.5588/ijtld.12.0476 23232005

[12] Lui G Wong RYK Li F Lee MKP Lai RWM Li TCM. High mortality in adults hospitalized for active tuberculosis in a low HIV prevalence setting. PLoS One. (2014) 9:e92077. doi: 10.1371/journal.pone.0092077, PMID: 24642794 PMC3958438

[13] Fløe A Hilberg O Wejse C Ibsen R Løkke A. Comorbidities, mortality and causes of death among patients with tuberculosis in Denmark 1998–2010: a nationwide, register-based case–control study. Thorax. (2018) 73:70–7. doi: 10.1136/thoraxjnl-2016-209240, PMID: 28778918

[14] Viana PVS Paiva NS Villela DAM Bastos LS Bierrenbach ALS Basta PC. Factors associated with death in patients with tuberculosis in Brazil: competing risks analysis. PLoS One. (2020) 15:e0240090. doi: 10.1371/journal.pone.0240090, PMID: 33031403 PMC7544107

[15] Shen X DeRiemer K Yuan Z Shen M Xia Z Gui X. Deaths among tuberculosis cases in Shanghai, China: who is at risk? BMC Infect Dis. (2009) 9:95. doi: 10.1186/1471-2334-9-95, PMID: 19531267 PMC2702371

[16] Mathew TA Ovsyanikova TN Shin SS Gelmanova I Balbuena DA Atwood S. Causes of death during tuberculosis treatment in Tomsk oblast, Russia. Int J Tuberc Lung Dis. (2006) 10:857–63. PMID: 16898369

[17] Min J Kim HW Ko Y Oh JY Kang JY Lee J. Tuberculosis surveillance and monitoring under the national public-private mix tuberculosis control project in South Korea 2016–2017. Tuberc Respir Dis. (2020) 83:218–7. doi: 10.4046/trd.2020.0016, PMID: 32610836 PMC7362746

[18] Moniruzzaman A Elwood R Wong H Kazanjian A FitzGerald J. A seventeen-year study of TB diagnosed postmortem in British Columbia, Canada. C106 Epidemiology of Mycobacterial Disease American Thoracic Society (2009). A5279.

[19] Lin CH Lin CJ Kuo YW Wang JY Hsu CL Chen JM. Tuberculosis mortality: patient characteristics and causes. BMC Infect Dis. (2014) 14:5. doi: 10.1186/1471-2334-14-5, PMID: 24387757 PMC3890594

[20] Min J Kim JS Kim HW Shin AY Koo HK Lee SS. Clinical profiles of early and tuberculosis-related mortality in South Korea between 2015 and 2017: a cross-sectional study. BMC Infect Dis. (2019) 19:735. doi: 10.1186/s12879-019-4365-9, PMID: 31438876 PMC6704578

[21] Sterling TR Zhao Z Khan A Chaisson RE Schluger N Mangura B. Mortality in a large tuberculosis treatment trial: modifiable and non-modifiable risk factors. Int J Tuberc Lung Dis. (2006) 10:542–9. PMID: 16704037

[22] Pradipta IS Boveneind-Vrubleuskaya NV Akkerman OW Alffenaar JWC Hak E. Predictors for treatment outcomes among patients with drug-susceptible tuberculosis in the Netherlands: a retrospective cohort study. Clin Microbiol Inf. (2019) 25:761.e1–7. doi: 10.1016/j.cmi.2018.10.009, PMID: 30394362

[23] Kourbatova EV Borodulin BE Borodulina EA Del Rio C Blumberg HM Leonard MK Jr. Risk factors for mortality among adult patients with newly diagnosed tuberculosis in Samara, Russia. Int J Tuberc Lung Dis. (2006) 10:1224–30. PMID: 17131780

[24] Zahar JR Azoulay E Klement E De Lassence A Lucet JC Regnier B. Delayed treatment contributes to mortality in ICU patients with severe active pulmonary tuberculosis and acute respiratory failure. Intensive Care Med. (2001) 27:513–20. doi: 10.1007/s001340000849, PMID: 11355119 PMC7095425

[25] Wang JY Hsueh PR Jan IS Lee LN Liaw YS Yang PC. The effect of smoking on tuberculosis: different patterns and poorer outcomes. Int J Tuberc Lung Dis. (2007) 11:143–9. PMID: 17263283

[26] Beadsworth MBJ van Oosterhout JJ Diver MJ Faragher EB Shenkin A Mwandumba HC. Hypoadrenalism is not associated with early mortality during tuberculosis treatment in Malawi. Int J Tuberc Lung Dis. (2008) 12:314–8. PMID: 18284838

[27] Hargreaves NJ Kadzakumanja O Phiri S Nyangulu DS Salaniponi FM Harries AD. What causes smear-negative pulmonary tuberculosis in Malawi, an area of high HIV seroprevalence? Int J Tuberc Lung Dis. (2001) 5:113–22. PMID: 11258504

