# Supplementary material for: Deaths from tuberculosis: differences between tuberculosis-related and non-tuberculosis-related deaths
Source: Front Public Health. 2023 Sep 1;11:1207284. doi: 10.3389/fpubh.2023.1207284 (PMC10502314; doi:10.3389/fpubh.2023.1207284)
Supplement: Supplementary file 1 [file Data_Sheet_1.docx]

Supplementary Material

Deaths from Tuberculosis: Differences between Tuberculosis-related and Non-tuberculosis-related Deaths

# Supplementary Tables

Supplementary Table S1. Predictive equation for the probability of death from (A) all causes, (B) TB-related causes, and (C) non-TB-related causes

(A) All cause death

$$\frac{e^{(-3.33+0.04\times Age-0.22\times Female-0.09\times BMI+0.24\times Alcohol+0.22\times Heart+0.50\times Liver+0.84\times Kidney+0.45\times CNS+1.27\times CNS inv+0.60\times Dyspnea+0.67\times GW-0.35\times Wt loss+0.33\times Bilat+0.35\times AFB smear)}}{(1+e^{\left( -3.33+0.04\times Age-0.22\times Female-0.09\times BMI+0.24\times Alcohol+0.22\times Heart+0.50\times Liver+0.84\times Kidney+0.45\times CNS+1.27\times CNS inv+0.60\times Dyspnea+0.67\times GW-0.35\times Wt loss+0.33\times Bilat+0.35\times AFB smear \right)})}$$

(B) TB-related death

$$\frac{e^{(-5.53+0.03\times Age-0.07\times BMI+0.75\times Dyspnea+0.46\times Fever+0.90\times GW+0.55\times Bilat+1.08\times AFB smear)}}{(1+e^{\left( -5.53+0.03\times Age-0.07\times BMI+0.75\times Dyspnea+0.46\times Fever+0.90\times GW+0.55\times Bilat+1.08\times AFB smear \right)})}$$

(C) Non-TB-related death

$$\frac{e^{(-3.43+0.04\times Age-0.32\times Female-0.09\times BMI+0.34\times Heart+0.76\times Liver+0.95\times Kidney+0.39\times CNS+1.05\times CNS inv+0.46\times Dyspnea+0.48\times GW-0.31\times Wt loss+0.23\times Bilat)}}{(1+e^{\left( -3.43+0.04\times Age-0.32\times Female-0.09\times BMI+0.34\times Heart+0.76\times Liver+0.95\times Kidney+0.39\times CNS+1.05\times CNS inv+0.46\times Dyspnea+0.48\times GW-0.31\times Wt loss+0.23\times Bilat \right)})}$$
